# Survey of attitudes toward performing and reflecting on required team service-learning (SASL): psychometric data and reliability/validity for healthcare professions students in preclinical courses

**DOI:** 10.3389/fmed.2023.1282199

**Published:** 2023-11-29

**Authors:** Lon J. Van Winkle, Shane L. Rogers, Bradley O. Thornock, Brian D. Schwartz, Alexis Horst, Jensen A. Fisher, Nicole Michels

**Affiliations:** ^1^Department of Medical Humanities, Rocky Vista University, Parker, CO, United States; ^2^Department of Biochemistry, Midwestern University, Downers Grove, IL, United States; ^3^School of Arts and Humanities, Edith Cowan University, Joondalup, WA, Australia; ^4^Department of Medical Humanities, Rocky Vista University, Ivins, UT, United States

**Keywords:** critical reflection, healthcare professions, preclinical courses, psychometric data, questionnaire validity, service-learning, student attitudes, survey reliability

## Abstract

**Purpose:**

Previously we assessed healthcare professional students’ feelings about team-based learning, implicit bias, and service to the community using an in-house paper survey. In this study, we determined whether this survey is a reliable and valid measure of prospective medical students’ attitudes toward required service-learning in an Immunology course. To our knowledge, no published questionnaire has been shown to be dependable and useful for measuring such attitudes using only eight survey items.

**Methods:**

Fifty-eight prospective medical students in Colorado (CO) and 15 in Utah (UT) completed the same Immunology course using remote technology. In addition to the usual course content, students were required to write critical reflections on required team service-learning. On the last day of class, they completed the survey of attitudes toward service-learning (SASL).

**Results:**

Data analyses found Cronbach’s alpha values of 0.84 and 0.85 for the surveys of UT and CO students, respectively. Factor analysis of CO student data revealed only one Eigenvalue greater than one (3.95) justifying retention of a single factor termed “attitudes toward required service-learning.” In addition, CO students’ attitudes toward community service were highly positive, while UT students’ attitudes were nearer neutral (*p* < 0.0001).

**Conclusion:**

Our factor analysis and good Cronbach’s alpha values support the conclusion that the SASL was a reliable measure of prospective medical students’ attitudes toward required team service-learning for an Immunology course. Moreover, we used the SASL to distinguish these attitudes in CO versus UT students, and, thus, the SASL appears to be a valid measure of this difference. Calculation of similarly good Cronbach’s alpha values – for a predecessor of the SASL among pharmacy, masters, and medical students at another institution – indicates that the SASL may be useful more widely. However, the reliability and validity of the SASL needs to be demonstrated more rigorously for other healthcare students at different universities.

## Introduction

Healthcare professional students’ cognitive empathy and compassion are fostered when they perform team-based service-learning (1–3). Moreover, written critical reflections on these experiences further promote students’ professional development (4–7). By exposing their implicit biases during service-learning, students are often moved to reflect on this dissonance, reconcile it, and, thus, work to avoid having it adversely affect their personal and professional behaviors (8, 9). Reconciliation of dissonance exposed by reflective practice is considered a primary mechanism to explain why such work can foster students’ cognitive empathy and compassion (8–12).

In this regard, writing critical reflections in teams on aspects of humanism in medicine for biochemistry courses has been found to foster patient-centered orientations in medical students, but this higher patient-centeredness lasts only as long as students are required to reflect (13). Likewise, cognitive empathy scores of medical students in biochemistry courses rose in association with reflections on service-learning, but only while students continued this work (14). Therefore, it has been argued that team-based work and reflective practice should continue throughout healthcare professionals’ careers to maintain their sense of compassion and help them to avoid burn-out (1, 2, 8, 15).

Despite evidence that indicates service-learning activities are beneficial, students may anticipate significant costs associated with participation that might act as a barrier for student engagement. For example, undergraduate college students felt they would have less time for their schoolwork (16) (*p* < 0.0001), and healthcare professions students have particularly heavy course loads during preclinical training. Such is also the case for prospective medical students hoping to gain access to our medical school by performing well, academically, in a Master of Science in Biological Sciences (MSBS) program. Therefore, to foster student engagement, there is a need to provide students with quantitative as well as qualitative evidence that students perceive value in service-learning activities.

A number of investigators have developed surveys to quantify students’ attitudes toward service-learning, but none focuses fully on students’ feelings about their experiences after performing community service (e.g., 16–18). Moreover, most such surveys measure attitudes in undergraduate college students, so there is a need to measure these attitudes among professional and graduate students who frequently have more demanding course workloads than undergraduates. Many of these other surveys are also quite demanding of students’ time, and may, sometimes, lead to survey fatigue.

For example, Shiarella and associates’ 46-item community service attitudes scale focuses more on undergraduate students’ expectations about the service rather than their experiences of it (16). Similarly, the 43-item civic attitudes and skills questionnaire of Moely et al. assesses students’ broader opinions about community service and social justice, but not their feelings about having participated in such service (17). While these attitudes, of students who may be planning to serve, are important, so are their feelings after they have done so.

In another 55-item survey, attitudes toward participating in service-learning by business and nonbusiness graduate and undergraduate students are measured, as are factors that encourage or discourage participation, but no evidence of the reliability or validity of the survey is reported (18). Service-learning has also been used to foster positive attitudes toward older adults in college and occupational therapy students (19, 20). But, again, neither of the latter papers report data supporting the reliability or validity of a survey concerning students’ attitudes toward service to the community. Other studies have focused on the ways in which service-learning helps to foster students’ professional development through improved humanistic characteristics, such as compassion, but they do not assess surveys of attitudes toward community service for reliability and validity (21–23). Finally, a 30-item questionnaire was used to assess service learning in the health professions, but this survey was designed for faculty and no data regarding reliability or validity were provided (24).

The aim of the present study is to develop more formally a much shorter 8-item questionnaire that we have used in prior research (8, 9, 11, 12, 14, 25). In that prior work, we used the survey to indicate healthcare professions students’ attitudes toward community service for preclinical basic science courses, but we did not attempt to determine the survey items’ collective reliability or validity. Hence, we tested two hypotheses concerning this questionnaire termed the survey of attitudes toward performing and reflecting on required team service-learning (SASL). Though designed for healthcare professions students, this short survey can, hypothetically, be used for any undergraduate or graduate course.

*Hypothesis 1:*The SASL is a *reliable* measure of students’ attitudes toward performing and reflecting on required team service-learning.

*Hypothesis 2:* The SASL is a *valid* measure of students’ attitudes toward performing and reflecting on required team service-learning.

## Methods

### Participants and team formation

Rocky Vista University Master of Science in Biological Sciences (MSBS) students participated in this study during their second semester of the MSBS program and as part of their Immunology course. Immunology was chosen for this study because the Immunology course directors were amenable to including service-learning in their course, while other preclinical course directors were not amenable. A major goal of these students was to perform well enough in the program to gain admission to our medical school. In this regard, 100% of MSBS graduates in this study achieved this goal, although the MSBS program also has an 8% attrition rate.

The Immunology course ran from January to May 2023. Fifty-eight MSBS students participated in the study in Parker, Colorado (CO), and 15 participated in Ivins, Utah (UT). In CO, 40 students (69%) identified as female and 18 (31%) were male. Additionally, 30 (52%) described themselves as White, while 10 (17%) said they were Asian, 9 (15%) were Black/African American, and 9 (15%) were Hispanic. Students in CO were between 21 and 37 years old (mean = 25.5 years old). In UT, 6 students identified as female (40%) and 9 (60%) were male. Also, 10 (67%) UT students said they were White, 2 (13%) were Asian, and 3 (20%) were Hispanic. Students in UT were between 22 and 44 years old (mean = 26.2 years old). In CO, teams of students were formed randomly on the first day of a Medical Humanities class in August of 2022 (first semester), and each team was comprised of six or seven students. Teams of three or four students were assigned in UT before the first day of the Immunology course in January 2023. Team service-learning projects were required as part of the course, and four written team and individual critical reflections on this community service, spaced throughout the semester, were assessed and contributed 10% of students’ grades in the course. Each student performed at least five hours of service usually with other members of their team. Teams were also expected to relate their service-learning to other Immunology course content.

Teams of students identified and selected their own community service projects for approval by one of us (LV). These projects in CO included service at homeless shelters, hospice and palliative care institutions, Special Olympics (and similar events), and Project Angel Heart (to feed immunocompromised people). In UT, students volunteered at food banks, helped maintain gardens to provide fresh food to community members, and worked with the Eagle Point ski patrol.

### Survey

Using our prior five-item survey of healthcare professions students’ attitudes toward selecting, performing, and reflecting on team service-learning projects (14), we modified and expanded the survey to include three more items concerning community service and two items to measure students’ opinions about their teammates (Table 1). CO and UT students completed this survey on the final day of their Immunology course. A faculty member in UT and an administrative assistant in CO, who were not involved directly in the course, distributed and collected the anonymous paper surveys and sent them to one of the authors (LV) for tabulation and analyses of the data. As secondary measures of students’ attitudes toward community service, we determined the mean word counts of their written critical reflections. And we report the results of university student evaluations of the instructor (LV) who assessed their critical reflections.

**Table 1:** Statistical comparisons of mean and standard deviation (SD) values of CO (*n* = 58) and UT (*n* = 15) students on individual items of the survey of attitudes toward performing and reflecting on required team service-learning (SASL)^a^.

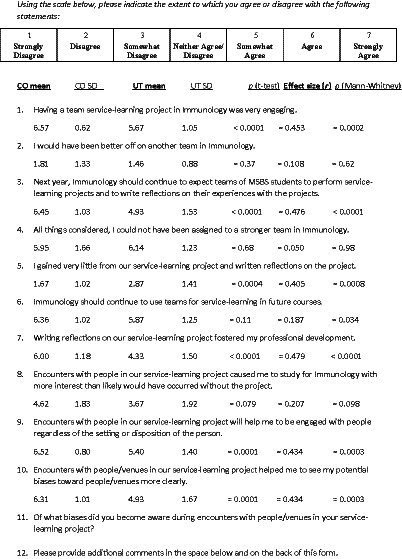

We also show results for nonparametric statistical comparison of median values, since many sets of data were skewed especially for CO students.

a Items 2 and 4 are also included in the table, although these items were not included in the final version of the survey, because they do not include the words “service-learning” or “community service” and instead concern students’ feelings about their teams.

This study (HIRB# 2018–0006) satisfies the criteria for exemption as determined by the Rocky Vista University Institutional Review Board (IRB). Informed consent was obtained from students to publish their written critical reflections.

### Statistical analyses

Cronbach’s alpha values were calculated using GraphPad Prism 9.5.1 Software Inc. (La Jolla, CA) for two-way analyses of variance yielding mean square values where Cronbach’s alpha = 1 – (residual mean square/row factor mean square). Pearson Intercorrelation values were calculated with the statistical program Stata, which was used for factor analysis of the survey items (26).

To begin to test the validity of the SASL, CO and UT students’ mean and median SASL scores and their mean and median responses to individual survey items were compared statistically using unpaired *t*-tests and nonparametric Mann–Whitney tests. The mean word counts of CO and UT students’ written reflections were also compared statistically using an unpaired *t*-test. When necessary, one-way analysis of variance and the Kruskal-Wallis test were used to compare more than two groups at a time, and the one sample *t*-test was employed to determine if the means of sets of data were significantly different from neutral. These latter statistical comparisons were made using the GraphPad Software.

## Results

*Hypothesis 1:* The SASL is a reliable measure of students’ attitudes toward performing and reflecting on required team service-learning.

Cronbach’s alpha values for the UT (*n* = 15), CO (*n* = 58), and combined (*n* = 73) student survey results at the end of the second semester Immunology course were 0.84, 0.85, and 0.88, respectively, demonstrating good survey reliability (100% response rates). In the first semester, Cronbach’s alpha was 0.84 for CO student responses at the end of their Medical Humanities course (*n* = 57, 98% response rate). For the five-item predecessor survey (14) on which the current survey was based, Cronbach alpha values for 191 medical, 104 masters, and 146 pharmacy student responses in biochemistry courses were 0.84, 0.88, and 0.90, respectively (new analyses of data obtained previously at another institution, (14, 25)).

Prior to conducting a factor analysis on one set of data (i.e., CO student responses in the second semester), the inter-correlations among the questionnaire items were obtained to check that items had at least an average moderate association with other items. As can be seen in Table 2, this was found to be the case. Items 2 and 4 were not included in our psychometric analyses of survey responses because they did not include the words “service-learning” or “community service.” Rather, these items focused more on students’ feelings about their teams (Table 1).

**Table 2:** Pearson Inter-correlations among the questionnaire items.

	Item 1	Item 3	Item 5	Item 6	Item 7	Item 8	Item 9	Item 10
Item 1	–							
Item 3	0.77	–						
Item 5	0.61	0.70	–					
Item 6	0.52	0.74	0.54	–				
Item 7	0.24	0.50	0.34	0.45	–			
Item 8	0.48	0.47	0.48	0.44	0.36	–		
Item 9	0.35	0.42	0.35	0.41	0.52	0.22	–	
Item 10	0.30	0.45	0.55	0.42	0.51	0.39	0.64	–
Average	0.47	0.58	0.51	0.50	0.42	0.41	0.47	0.47

An exploratory factor analysis was performed using the statistical program Stata (26) for CO students at the end of their Immunology course. More specifically, we used the default ‘principal factor’ method in Stata that analyses the common variance, instead of the total variance which is analysed via principal components analysis. We took this approach as we are examining the factor structure of this questionnaire for the first time. Only a single Eigenvalue was greater than 1 (i.e., an Eigenvalue of 3.95, equating to 85% variance explained) justifying the retention of a single factor. The factor loadings are all greater than the typical 0.4 cut-off (Table 3). In Table 3 the factor loadings represent the simple correlation between each item and the underlying factor. The uniqueness value represents the variance that is not shared with other variables (it is equal to 1 – communality). For example, 27% of the variance in item 1 is not shared with other variables in the overall factor model. If uniqueness is relatively high (e.g., over 0.60), then the item can be questioned as fitting well within the overall set of items. We provide uniqueness values alongside the factor loadings as they provide complementary additional information for evaluating the quality of items. The Cronbach’s alpha of the questionnaire for this set of data across all eight items was 0.85. These results provide evidence for the reliability of the SASL.

**Table 3:** Factor loadings and uniqueness values from exploratory factor analysis of the Survey of Attitudes Toward Performing and Reflecting on Team Service-learning (SASL).

	Factor loading	Uniqueness value
1. Having a team service-learning project in (name of course) was very engaging.	0.73	0.27
3. Next year, (name of course) should continue to expect teams of MSBS students to perform service-learning projects and to write reflections on their experiences with the projects.	0.88	0.13
5. I gained very little from our service-learning project and written reflections on the project. (Reverse scored)	0.75	0.35
6. (name of course) should continue to use teams for service-learning in future courses.	0.75	0.39
7. Writing reflections on our service-learning project fostered my professional development.	0.60	0.47
8. Encounters with people in our service-learning project caused me to study for (name of course) with more interest than likely would have occurred without the project.	0.58	0.58
9. Encounters with people in our service-learning project will help me to be engaged with people regardless of the setting or disposition of the person.	0.60	0.41
10. Encounters with people/venues in our service-learning project helped me to see my potential biases toward people/venues more clearly.	0.67	0.34

*Hypothesis 2:* The SASL is a valid measure of students’ attitudes toward performing and reflecting on required team service-learning.

**Figure 1:**
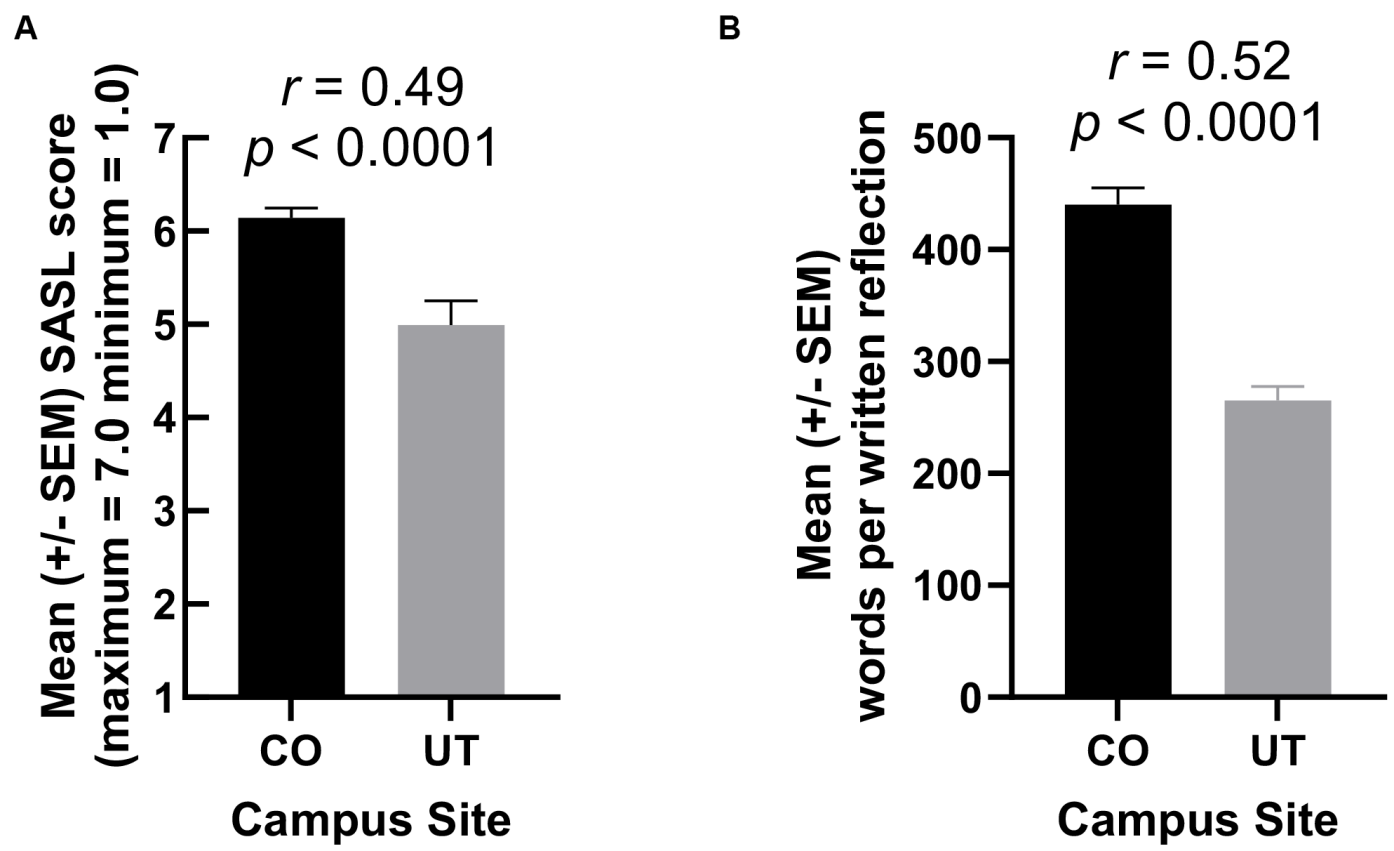
**(A)** Mean CO and UT student scores on the survey of attitudes toward performing and reflecting on required team service-learning (SASL). **(B)** Mean words per written reflection by CO and UT students concerning their team service-learning projects for their Immunology course.

A comparison of the two student groups in Immunology was conducted to provide some preliminary evidence for the utility and validity of the survey. The mean score on the survey for CO students was significantly higher than for UT students (Figure 1A, *t* (71) = 4.79, *p* < 0.0001), and this difference was of near crucial practical importance (*r* = 0.49) (27). That is, an *r* value of 0.49 has been reported to be equivalent to treatment by a drug that reduces the death rate from 74.5 to 25.5% - difference equals 49% (28). Also, the mean score of CO students in Immunology (Figure 1A) was statistically indistinguishable from their prior mean score at the end of their first semester Medical Humanities course (prior mean = 6.25, *p* = 0.42). The learning objectives for the service-learning component of Immunology were the same as this component in Medical Humanities. The stability of CO students’ SASL scores across the second semester of the MSBS program supports the notion that the survey is valid and reliable (17), although correlation analyses were not possible because surveys were anonymous.

The survey items and the mean responses to each item by CO and UT students are also shown in Table 1 for service learning in the second semester of the MSBS program. Similar means were observed for CO students in the first semester when the course name in Table 1 items was “Medical Humanities” instead of “Immunology” (data not shown). Except for items 2 and 4, the mean responses of UT students were closer to neutral than for CO students as also indicated by their overall SASL scores for their attitudes toward performing and reflecting on team service-learning (Figure 1A). For example, even in the case of item 8 (Table 1), the mean value for CO students (4.62) was significantly greater than neutral (i.e., 4.00, *p* = 0.01), whereas this mean for UT students (3.67) was statistically indistinguishable from neutral (*p* = 0.51).

Moreover, in association and in keeping with their less enthusiastic attitudes toward required service-learning for their Immunology course (Table 1; Figure 1A), UT students’ written reflections concerning service-learning were considerably shorter than those of CO students (Figure 1B, *t* (166) = 7.87, *p* < 0.0001), and this difference was of crucial practical importance (*r* = 0.52) (27). Consistent with the latter finding, UT students exhibited less *critical* reflection than CO students in their final written reflections (*p* < 0.0001). It is, however, also conceivable that, in the latter case, the author who assessed the reflections (LV) was biased in favor of CO students with whom he had formed relationships during the first semester of the MSBS program (See results). We define *critical* reflection as the extent to which students describe their self-examination and compassionate behavior in their written reflections, as discussed by us elsewhere (9).

CO and UT students could also be distinguished by their answers to questions 11 and 12 of the survey (Table 1). Although most students did not respond to question 12, those CO students who did answers question 12 were much more positive than UT students (Chi-square = 7.36, *p* < 0.01). In CO, students who answered usually said “loved service-learning” (or something similar), while UT students who replied were more likely to give responses like “service-learning is out of place in Immunology.” And, while not statistically significant (Chi-square = 2.51, *p* = 0.11), CO students answered question 11 more frequently (46 of 62 responses) than did UT students (8 of 15 responses) (Table 4). According to all the results, the SASL appears to be a valid as well as reliable measure of healthcare professions students’ attitudes toward selecting and performing team community service projects in preclinical courses.

**Table 4:** Summary of written statements of biases expressed by students in the survey about their team- and service-learning experiences (42 of 58 CO and 8 of 15 UT students stated one or more of their biases in response to question 11 in Table 1).

Nature of negative biases in CO	Number (%) of times expressed	Nature of negative biases in UT	Number (%) of times expressed
Homeless people	11 (17.5)	Homeless people	3 (20.0)
Proximity bias	9 (14.3)	Proximity bias	2 (13.3)
English 2^nd^ language	4 (6.3)		
Hospice/palliative care	4 (6.3)		
Special needs people	3 (4.8)		
Research bias	2 (3.2)		
Privileged Americans	2 (3.2)	Privileged Americans	1 (6.7)
Premature judgment	1 (1.6)		
Alcohol/drug addiction	1 (1.6)		
Poverty prevalence	1 (1.6)		
Immune complications	1 (1.6)		
Dying people	1 (1.6)		
Going outside comfort	1 (1.6)		
Expecting thanks	1 (1.6)		
Speaking not listening	1 (1.6)		
Not caring for self	1 (1.6)		
Criminal background	1 (1.6)		
Older people	1 (1.6)		
		Volunteering	2 (13.3)
No answer	16 (25.4)	No answer	7 (46.6)

## Discussion

The purpose of our study was to develop more formally a short 8-item questionnaire – now termed the survey of attitudes toward performing and reflecting on required team service-learning (SASL) – that we have used in prior research (8, 9, 11, 12, 14, 25). In our prior work, however, we did not attempt to determine the survey items’ collective reliability or validity. Here we used factor analysis to formally assess SASL’s reliability (our hypothesis 1), and we provide evidence for its validity (our hypothesis 2).

### Reliability and validity of the SASL

The exploratory factor analysis of the SASL for responses of 58 CO students in their Immunology course revealed a single Eigenvalue greater than one justifying a single factor we call “attitudes toward required service-learning” for the SASL (Table 3). A good Cronbach’s alpha value of 0.85 for the same set of data provides further evidence of the reliability of the SASL. And the Cronbach’s alpha value of 0.88 for all 73 CO and UT MSBS students in Immunology during the spring semester of 2023 further supports this conclusion. While further testing of SASL’s reliability is needed among other healthcare professions students and college students more broadly, reassessment of prior data for the predecessor of the SASL indicates that the SASL will be found to be more widely reliable. That is, this five-item precursor survey administered to 191 medical, 104 masters, and 146 pharmacy students in biochemistry courses yielded Cronbach’s alpha values of 0.84, 0.88, and 0.90, respectively (new analysis of data obtained previously at another institution, 11, 25). Hence, we feel the SASL is likely a reliable indicator of how health professions students feel about their required service-learning experiences in preclinical basic sciences courses.

In addition, we provide preliminary data that the SASL is useful and valid for detecting differences in attitudes toward required service-learning when students’ experiences vary. UT students were much closer to neutral than CO students in their attitudes toward community service both according to the SASL (Figure 1A) and individual items on the survey (Table 1). While the population sizes of these two groups are quite different (i.e., 15 in UT vs. 58 in CO), the magnitude and effect sizes for their differences are profound. For example, according to item 7 “Writing reflections on our service-learning project fostered my professional development,” the mean UT response of 4.33 was indistinguishable from the neutral value of 4.00 (GraphPad one sample *t*-test, *t* (14) = 0.86, *p* = 0.40), while the mean of 6.00 for CO students was well above neutral (one sample *t*-test, *t*(57) = 12.86, *p* < 0.0001). And the effect size for CO students (*r* = 0.86) was much greater than the minimum of 0.50 required for the label “crucial practical importance” (27).

### From where might the difference in attitudes toward service-learning have arisen in CO versus UT students?

Several factors may have contributed to the differences in CO and UT students’ attitudes toward community service in their Immunology course. For example, CO students had a greater proportion of women students, and women seem to have a more positive view of service-learning than do men (18). However, the main reason for the difference between CO and UT student scores seems to us to be the relationships of the students to the faculty member grading their critical reflections on service-learning experiences. To be effective at promoting personal growth and professional development, students need to embrace the vulnerability they feel in writing these personal reflections (8, 29). Having formed good relationships with students as Director of their Medical Humanities course during the first semester of the MSBS program, one of us (LV) provided a safe space for teams of CO students to exhibit this vulnerability in their written reflections for Immunology.

For example, CO students’ rating of LV on the university course evaluation form for Medical Humanities averaged 4.7 (five-point Likert scale) for the item “This faculty member effectively helped me to learn the course material and contributed to my development as an active, independent learner.” And this rating was described by the university as “much higher” than the average ratings of other faculty members teaching, specifically, in the MSBS program, as well as in all preclinical courses at the university. In keeping with this result, LV’s rating by CO students on the same item for Immunology in the second semester averaged 4.8, which was also “much higher” than the average for other faculty members. In contrast, the average rating of LV on the same item by UT students was 3.8 and “lower” than other faculty members at the university. But LV had little opportunity to form relationships with UT students in the first-semester Medical Humanities course, and UT students and LV had no opportunity to get to know and trust one another on a personal level in Immunology in the second semester. These differences in the ratings of LV by CO students in the first and second semester and by UT students in the second semester were highly statistically significant (Kruskal-Wallis test for these three sets of data, *p* < 0.0001). And the effect size of the differences (*r* = 0.58) was estimated to be of crucial practical importance (27).

To attempt to improve UT students’ attitudes toward service learning in Immunology in future years, another of us (BT) will assess UT students individual and team reflections concerning service-learning experiences. BT directed the Medical Humanities course in UT during the first semester of the MSBS program and, so, was able to form trusting relationships with UT students. This trust was built, in part, owing to his assessment of written critical reflections by students on related experiences in UT Medical Humanities meant to elicit students’ dissonance, vulnerability, and resolution of this dissonance (8) without service-learning. In support of these possibilities, BT’s average rating, for the UT Medical Humanities course and on the university course evaluation item discussed above, was 4.6, which was also “much higher” than the average rating of other faculty members teaching preclinical courses at the university.

Similarly, when BT and LV cotaught Medical Humanities remotely in 2020, and LJV assessed critical reflections on service-learning for both CO and UT students, both BT and LV received average ratings of 4.6 on the above survey item by both CO and UT students. Then, after forming good and trusting relationships with both groups of students in Humanities, LV received average ratings of 4.6 from both groups of students for assessing their reflections on community service for the subsequent remote Immunology course in 2021. Hence, we believe the strategy above, to have BT assess reflections on service-learning by UT students for Immunology, will significantly improve UT students SASL scores in future years.

More broadly – for faculty members in other courses and at different institutions – attempts to promote students’ personal growth and professional development through critical reflection on service-learning should involve forming trusting relationships between the students and faculty members. We believe, only then, will students embrace the vulnerability they feel in reflecting on and writing these personal reflections and, thus, foster their compassion and professional development (8, 29).

### Use of community service to foster other aspects of professional development: impact of service-learning

Around the world (30–32), and particularly in the US (10, 33–41) the issue of implicit bias remains poorly controlled. For example, negative attitudes toward people of color and others result in discrimination in their healthcare by those expected to deliver it (39–41). But one way to foster bias mitigation through critical reflection on team community service has been shown by us also to promote empathy and compassion in prospective medical students (8, 9). Such training efforts have potential to be applied in other healthcare professions (39, 41). These curricula would foster the health of those people against whom there are unconscious biases and, thus, promote public health (42).

Similarly, other investigators have used “a pedagogy of discomfort” (32, 33), and the “transformative learning model” of Sukhera et al. (36) parallels our methods and findings. Like us (8, 9, 11, 12, 14), Sukhera and associates initiate experiences that produce dissonance by uncovering students’ implicit biases. These experiences then lead to self-examination and critical reflection by the students. This reflection then often fosters behaviors such as listening to people’s stories with genuine interest and compassion. Many other recent publications also make strong cases to promote and maintain healthcare professions students’ empathy and compassion through sustainable curricula (e.g., (10, 37, 38)), and such results can be achieved using reflection on service to the community (3, 29).

## Limitations/Conclusions

Our study was limited to 73 prospective medical students in a second-semester Immunology course at a single university. Nevertheless, Cronbach’s alpha for CO students (n = 58) remained stable for the SASL near a good 0.85 value in the first as well as in the second semester. This value was also near 0.85 when calculated for the population of just 15 UT students. Moreover, a factor analysis, with a single Eigenvalue of 3.95, strongly justified retention of a single SASL factor we termed “attitudes toward required service-learning.” These results provide evidence for the reliability of the SASL. While our current studies were limited to MSBS students alone, reassessment of previously obtained data concerning a predecessor survey to the SASL supports the notion that the SASL will be found to be more widely reliable. Using those prior data for that five-item survey, we calculated Cronbach’s alpha values of 0.84, 0.88, and 0.90 for 191 medical, 104, masters, and 146 pharmacy students, respectively, in Biochemistry courses at another university (14, 25).

Our data also indicate that the SASL is valid and useful for detecting differences between populations of students with different experiences of community service-related activities, at least at our institution. For example, UT students’ attitudes were nearer neutral toward performing team service-learning, while the attitudes of CO students were highly positive (Table 1; Figure 1). We attribute these large differences to two main factors. Firstly, CO students became accustomed to performing service-learning in their Medical Humanities course in the semester preceding their Immunology course, whereas UT students did not. And we think, more importantly, UT students had no opportunity to build trusting relationships with the faculty member assessing their reflections about their service-learning experiences – and who was likely seen as requiring the service in the first place. In contrast, CO students formed relationships with this faculty member as Director of their Medical Humanities course during the first semester of the MSBS program. These trusting relationships are, in our view, essential since students’ critical reflections on team community service experiences become very personal and reveal their vulnerabilities as well as strengths in coping, productively, with the dissonance evoked through service-learning and related activities (8, 29). Although we believe the SASL will prove valid in identifying other such experiential differences of service-learning in different groups of students at other universities, the extent of this SASL utility remains to be established.

Similarly, it would be ideal to perform follow up studies with the participants in our study, not only to survey them, but also to implement curricular interventions aimed at maintaining positive attitudes toward service-learning during their continued training in medical school. In this regard, the first semester inter-professional education (IPE) course for first-year medical and physician assistant students at our institution does include critical reflection on team community service. Unfortunately, however, this IPE course is the only one that requires service-learning experiences in the entire curricula of those programs. Hence, we, as well as most other healthcare training programs, should foster student compassion by including regular critical reflection on such experiences throughout our curricula.

## Data availability statement

The original contributions presented in the study are included in the article/supplementary material, further inquiries can be directed to the corresponding author.

## Ethics statement

The studies involving humans were approved by this study (HIRB# 2018-0006) satisfies the criteria for exemption as determined by the Rocky Vista University Institutional Review Board (IRB). Informed consent was obtained from students to publish their written critical reflections. The studies were conducted in accordance with the local legislation and institutional requirements. The participants provided their written informed consent to participate in this study.

## Author contributions

LW: Conceptualization, Data curation, Formal analysis, Investigation, Methodology, Project administration, Validation, Writing – original draft, Writing – review & editing. SR: Conceptualization, Data curation, Formal analysis, Investigation, Methodology, Validation, Writing – original draft, Writing – review & editing. BT: Conceptualization, Writing – review & editing. BS: Conceptualization, Writing – review & editing. AH: Conceptualization, Writing – review & editing. JF: Conceptualization, Writing – review & editing. NM: Conceptualization, Writing – review & editing.
